# The configuration path of the balanced compulsory education resources supply in the context of equal rights to rent and purchase: Based on the fsQCA analysis of 31 cities in China

**DOI:** 10.1371/journal.pone.0308286

**Published:** 2024-09-06

**Authors:** Wenlong Lou, Jinjie Li

**Affiliations:** 1 School of Public Management, Yanshan University, Qinhuangdao, Hebei, China; 2 Key Research Bases of Humanities and Social Sciences for Universities in Hebei Province: Yanshan University County Area Revitalization Development Policy Research Centre, Qinhuangdao, Hebei, China; Universidad de Murcia Faculty of Economics and Business: Universidad de Murcia Facultad de Economia y Empresa, SPAIN

## Abstract

Equal rights to rent and purchase affects the supply of compulsory education resources. How to promote the balanced compulsory education resources supply in the context of equal rights to rent and purchase is currently a hot issue amongst government and society. To achieve such balance, conducting research in the context of equal rights to rent and purchase becomes crucial. However, existing research has yet to provide sufficient explanations for the differentiated paths for realizing the balanced compulsory education resources supply in practice. This study uses 31 cities in China as case samples and jointly applies fuzzy-set qualitative comparative analysis and the ‘technology–organization–environment’ (TOE) framework. The findings are summarized as follows. Firstly, the balanced compulsory education resources supply in the context of equal rights to rent and purchase is affected by six key technological, organizational, and environmental factors. Namely, data co-construction and sharing, technological infrastructure, attention allocation, government information disclosure, policy support for equal rights to rent and purchase, and level of urban economic development. Secondly, the linkage matching of technological, organizational and environmental conditions forms diversified configurations that drive the balanced compulsory education resources supply in the context of equal rights to rent and purchase. These configurations include the ‘organization’ driving model, ‘technology–environment’ driving model, ‘technology–organization–environment’ driving model, and ‘technology-organization’ driving model. Finally, eastern, central and western China are driven by different configuration paths. Amongst them, eastern China has relatively good basic conditions at the technological, organizational and environmental levels. The combination of different conditions can drive the balanced compulsory education resources supply in a ‘different paths lead to the same destination’ manner. Although the development in central China is somewhat restricted, the radiation and impetus from eastern China, in combination with the different conditions in central China, can drive the balanced compulsory education resources supply. Western China shows no advantages at the technological, organizational and environmental levels. Faced with restrictions in organizational and environmental conditions, the government in western China should develop the necessary technological conditions to drive the balanced compulsory education resources supply.

## 1 Introduction

‘Coordinated development of renting and purchasing’ represents a new direction for the future development of the housing system reform in China [1] as clearly stated by the central government of China in recent years. ‘Equal rights to rent and purchase’ has been proposed as an important policy guidance to promote ‘the coordinated development of renting and purchasing’. In 2017, the report of the 19th National Congress of the Communist Party of China proposed ‘accelerating the establishment of a housing system with multiple entities supplying, multiple channels guaranteeing, and coordinated development of renting and purchasing.’ It identifies ‘the coordinated development of renting and purchasing’ as a new direction for the reform and development of the housing system in China. In 2017, the General Office of Guangzhou Municipal People’s Government in China issued the ‘Work Plan for Accelerating the Development of the Housing Rental Market in Guangzhou’ (Document No.29 of the Guangzhou Municipal Government Office [2017]). It explicitly states that ‘Granting eligible tenants’ children the right to access public services, such as nearby enrollment, ensuring equal rights to rent and purchase.’ Following the issuance of this plan, Guangzhou became the first city in China to introduce a new policy on housing rental and to propose and implement ‘equal rights to rent and purchase’. Subsequently, other pilot cities for housing rental reforms have issued relevant work plans, which reflect the goal orientation of ‘equal rights to rent and purchase’ to varying degrees [2].

The above advancements suggest that the housing rental and housing sales markets in China have not yet formed a balanced development as a whole [3]. In addition, significant differences can be observed between the treatment of the renting and purchasing groups in terms of access to public services [4].

The focus of social concern over equal rights to rent and purchase mainly rests on children’s right to education. Equal rights to rent and purchase decouples access to public services, including children’s education, health care and elderly care, from housing ownership, thus enabling the renting and purchasing groups to enjoy these rights on an equal footing. Therefore, since its announcement, equal rights to rent and purchase has been widely acclaimed by the public. It is particularly welcomed by young and middle-aged people in the middle class who cannot afford their own houses and thus have no access to public education resources for their children [5]. Despite the high public expectation, the implementation effect of equal rights to rent and purchase has not been as effective as expected [6]. For instance, the ‘equal rights’ implemented in pilot cities only guarantees that the tenants’ children have the right to access compulsory education in the city, but does not guarantee access to quality schools nearby. In many cities, even homeowners in matching districts have not guaranteed to access quality compulsory education resources. Several measures, such as waiting lists or quotas based on time limits, are often implemented in these areas. Therefore, tenants are unable to access compulsory education resources as easily as homeowners do. This phenomenon can be observed even in pilot cities that implement equal rights to rent and purchase. Based on these arguments, the advocacy of equal rights to rent and purchase is more symbolic than practical despite its positive significance [7].

Faced with the opportunities and challenges in achieving the balanced compulsory education resources supply in the context of equal rights to rent and purchase, local governments in China have accelerated reforms in the housing and education sectors. The Chinese government has taken several measures to promote the housing reform, including cultivating and developing the housing rental market to compensate for the structural deficiencies in its urban housing supply [8]. Meanwhile, the right to access compulsory education resources should be appropriately separated from the right to housing. For example, the implementation of cross-district teacher exchange and rotation in the urban areas of Beijing has alleviated the housing and education bundling issue represented by ‘school district housing’ to some extent. The implementation of the large school districts enrollment model in Shenzhen aims to achieve the sharing and utilization of quality compulsory education resources within the regional spatial scope. This model also seeks to avoid the skyrocketing housing prices and rents caused by speculative leasing competition for quality compulsory education resources. These policies aim to break the link between housing and compulsory education and promote a gradual shift from an unbalanced quality compulsory education resources supply to a balanced compulsory education resources supply. Many practices show that local governments in China are making efforts to separate the right to access compulsory education resources from the right to housing. However, this problem cannot be solved in the short term, and China still has a long way to go to realize equal rights to rent and purchase thoroughly. Based on the above statements, this study aims to answer several questions. Firstly, which key factors affect the balanced compulsory education resources supply in the context of equal rights to rent and purchase? And what are the complex interactions of these key influencing factors and the configuration paths they generate? Secondly, which differentiated configuration paths have local governments in eastern, central and western China used to drive their balanced compulsory education resources supply? Thirdly, what is the significance of the identified differentiated configuration paths for the future policy implementation precisely by local governments in China?

This paper selects 31 representative cities from 31 provinces (autonomous regions and municipalities directly under the central government of China) of China as case samples and applies the fuzzy-set qualitative comparative analysis (fsQCA) method for the case study. The contributions of this study mainly cover three aspects. First of all, this study fills the research gap of existing studies and makes a theoretical contribution. Firstly, previous research has mostly investigated the supply of compulsory education resources in the context of equal rights to rent and purchase via theoretical analyses and policy commentaries but has failed to identify those factors that influence such supply. This paper fills such gap to some extent by focusing on 31 cities in 31 provinces where equal rights to rent and purchase have a relatively mature implementation. From the perspective of ‘technology-organization-environment’ (TOE), this study identifies six key factors that affect the balanced compulsory education resources supply in the context of equal rights to rent and purchase. Secondly, previous studies have not identified the differentiated paths adopted by the Chinese local governments to drive the balanced compulsory education resources supply. To fill such gap, this paper gives full play to the advantages of the configuration method in the case study and proposes several models for driving the balanced compulsory education resources supply. Furthermore, this study contributes to the understanding of the balanced compulsory education resources supply in the context of equal rights to rent and purchase from multiple levels, perspectives and regions. Specifically, this research offers insights into the core influencing factors, their complex interactions and configuration paths from the multiple levels of technology, organization and environment, multiple driving models and multiple regions of eastern, central and western China. Finally, by applying fsQCA, this study offers a key methodological contribution to related research. The fsQCA method holds that the antecedent conditions will collaboratively achieve the results with diverse configuration types [9]. The differentiated configuration paths identified in this study hold great significance that would help local governments in China implement the future policies precisely.

## 2 Literature review

### 2.1 Development course and implementation status of equal rights to rent and purchase

#### 2.1.1 Development course of equal rights to rent and purchase

To alleviate the social inequality issues stemming from unequal access to education, many countries have formulated various policies, such as school district consolidation in the United States, inter-regional enrollment in the United Kingdom and elimination of school districts in Norway [10, 11]. Decoupling education from housing is a problem faced by many countries. Since the reform and opening up in China, the urbanization process has been accelerating, thus driving people with rural household registrations to move into cities. The pressure on urban public service resources such as education resources, housing resources and medical resources has intensified in turn. Amongst these resources, housing is particularly important for rural migrants, because "settling down" makes "working with joy" possible. Housing is related not only to the quality of urbanization but also to basic livelihood [12]. However, the vast majority of the rural migrant workers have low housing affordability, thus limiting their options to renting housing [13]. The long-standing emphasis on selling rather than renting in the housing supply sector of China has led to some issues in the housing rental market and in the social rights security of renters. Firstly, the demand for rental housing in large and medium-sized cities is particularly high given their large influx of rural migrants, thus resulting in structural contradictions between housing rental supply and demand. Secondly, the housing rental market lacks regulations and offers inadequate protection to the basic rights of renters [14]. Thirdly, a series of institutional arrangements unique to China, such as its household registration, social security and school district systems, objectively limit the access of urban renters to social rights [15]. Significant differences can be observed between renters and homeowners in terms of access to public services. To address these problems, in 2015, the Central Economic Work Conference of China proposed ‘taking the establishment of a housing system with the coordinated development of renting and purchasing as the main direction’. In 2016, the General Office of the State Council of China issued ‘Several Opinions on Accelerating the Cultivation and Development of the Housing Rental Market’ (Document No.39 of the General Office of the State Council of China [2016]). This document encouraged local governments to accelerate the development of the housing rental market according to local conditions. In 2017, the Ministry of Housing and Urban–Rural Development of the People’s Republic of China and nine other departments issued the ‘Notice on Accelerating the Development of the Housing Rental Market in Large- and Medium-Sized Cities with Net Population Inflows’ (Document No.153 of the Ministry of Housing and Urban–Rural Development of China [2017]). It selected 12 cities, including Guangzhou, Shenzhen and Wuhan, as the first batch of cities to launch housing rental pilot programmes. These policies and documents gradually promoted the development of the housing rental market in China towards standardization, institutionalization and normalization. Local governments of China have also successively introduced new housing rental policies to promote the development of the housing rental market. In 2017, the General Office of Guangzhou Municipal People’s Government in China issued the ‘Work Plan for Accelerating the Development of the Housing Rental Market in Guangzhou’ (Document No.29 of the Guangzhou Municipal Government Office [2017]). It makes Guangzhou the first city to introduce a new policy on housing rental and to implement ‘equal rights to rent and purchase’. Since then, other pilot cities for housing rental reforms have successively issued their work plans to promote equal rights to rent and purchase. For example, in 2017, Nanjing issued the ‘Nanjing Pilot Work Plan for Housing Rental’. It explicitly stated that equal rights to rent and purchase would be gradually realized in several fields, such as compulsory education, basic medical care, housing security and provident fund withdrawal.

#### 2.1.2 The implementation status of equal rights to rent and purchase

The existing literature shows that people can choose their place of residence and corresponding public services according to their income and preferences. Therefore, people’s choices affect the quality differences in public services that are capitalized in house prices [16–18]. In China, from the overall perspective of policy design, the starting point of equal rights to rent and purchase is to grant the renting and purchasing groups equal access to public services. Although this goal has not yet been realized, the Chinese governments at all levels hold the view that only by implementing equal rights to rent and purchase can they enhance the substitutability of renting for purchasing [19]. Thus the fairness of public services can be improved.

The implementation of equal rights to rent and purchase still faces certain practical constraints. Such as funding and housing supply issues, poor-quality public service resources, lack of relevant regulatory agencies and the long-standing concept of ‘house purchasing for settling down’ [20]. The implementation has been slow in various places. These constraints also explain the weak development of the housing rental market and prevent the renting and purchasing groups from having equal access to public services. To address these problems, the development of equal rights to rent and purchase should follow the principle of gradual progress. Specifically, this policy guidance should be promoted in an orderly manner by type and level, overcoming the difficulties gradually in the policy implementation process. It also should gradually move from single and basic equal rights to multiple and comprehensive equal rights [21].

### 2.2 Research progress on equal rights to rent and purchase and compulsory education resources supply

#### 2.2.1 Research progress on equal rights to rent and purchase at the level of access to public services

Existing research has mainly divided the connotation of equal rights of renting and purchasing into two levels. Firstly, the significance of equal rights to rent and purchase is to break the rights systems of education, medical care and pension from the housing property rights [22]. It aims to restore the public nature of public resources [23]. Whilst a unified and official definition of ‘rights’ in ‘equal rights of renting and purchasing’ is yet to be proposed. The Ministry of Housing and Urban–Rural Development of China claimed that the new legislation will be introduced. The new legislation will clarify ‘equal rights to rent and purchase’ as ‘enabling tenants to enjoy equal treatment with homeowners in basic public services’ [24]. The new legislation suggests that the government is starting to face up to the problem of unfair distribution caused by the scarcity of public resources. Secondly, equal rights to rent and purchase points towards the equality of access to public services. It also emphasizes the government’s extensive and far-reaching social reforms and management approaches innovation in the pilot and policy implementations of equal rights to rent and purchase [25]. Existing research on the second connotation have examined the relationship between the right to housing and the right to access public services in the context of equal rights to rent and purchase from a broader perspective. It emphasizes that through the governance reform of the government and society, equal rights to rent and purchase can promote the equalization of public services in the whole society. Instead of focusing on the ‘right’ in the ‘equal rights to rent and purchase’, this connotation focuses on innovative governance in addressing various complex problems in social development.

Existing studies on equal rights to rent and purchase have mainly focused on the single and comprehensive perspectives, such as market equal subjects, institutional innovation and residents’ well-being. The perspective of market equal subjects explores how the government empowers the relatively disadvantaged groups, improving the unfavorable situation of renters and highlighting the characteristics of non-discriminatory access to public services [23]. The perspective of institutional innovation mainly analyzes how equal rights to rent and purchase break down the barriers between housing and public resources, such as education resources. And how this process drives the reform of public resources allocation, promotes governance innovation and facilitates the formulation of public policies [14]. The perspective of residents’ well-being mainly explores whether equal rights to rent and purchase can improve the subjective well-being of the renting group. That is, whether the transformation of the right to access public services from being linked to housing property rights to being linked to the fundamental housing rights can improve the well-being of renters [26].

#### 2.2.2 Research progress on equal rights to rent and purchase at the level of compulsory education resource supply

Firstly, previous research has explored the necessity and urgency of implementing equal rights to rent and purchase at the level of compulsory education resources supply. The scarcity and unequal distribution of quality education resources hinder improvements in fairness of access to education [27–29]. Many countries around the world, such as the United Kingdom [30], United States [31, 32] and South Korea [33], attempt to improve the fairness of distribution of quality education resources by specifying enrollment nearby or school district divisions. However, such division of school districts leads to differences in access to quality compulsory education and triggers a competition for school district housing [34]. Scholars have defined the social inequality caused by the availability of education [35, 36], and the premium on high housing prices in school districts offering quality education [37, 38]. Due to the household registration system, housing property ownership and other factors in China, children of migrant workers are deprived from accessing the same compulsory education resources enjoyed by residents with urban household registrations [39]. Meanwhile, the realistic hardship of compulsory education enrollment nearby based on household registration urgently needs to be addressed. The issue of "school district housing" has pushed the fairness of compulsory education to the limelight. Therefore, promoting the balanced development of compulsory education is an important task to alleviate the structural contradiction on the supply side of compulsory education. Equal rights to rent and purchase is a mean for the government to promote the fairness of compulsory education.

Secondly, previous research has explored the discrepancies in the right to education between the renting group and the purchasing group. Existing studies have mainly focused on unequal rights to enrollment, the causes of such inequality and the impact of equal rights to rent and purchase on the well-being of the renting and purchasing groups. In Japan and some countries in Europe and America, the renting and purchasing groups enjoy equal access to enrollment. And the quality of schools is highly positively correlated with housing rent [40]. Under the current policy of tying housing to school districts in China, the children of the renting and purchasing groups enjoy different qualities of compulsory education due to spatial differences in housing. The quality of compulsory education schools is the competitiveness of the school district [41]. Through theoretical deduction, Chinese scholars held that equal rights to rent and purchase will lower the threshold for low and middle-income groups to access quality education resources [19]. Whilst the inequality in the right to enrollment has many complex drivers, this study only focuses on housing-related causes. Due to unequal rights to rent and purchase, quality compulsory education resources in China are capitalized in housing prices [42]. The capitalization of quality compulsory education resources has a crowding-out effect on low- and middle-income groups. It will persist in the short term after the implementation of equal rights to rent and purchase. Therefore, equal rights to rent and purchase play a guiding role in addressing this problem. Specifically, this policy guidance aims to avoid the potential risk of shifting from renting for ‘living’ to renting for ‘school district enrollment rights’. Meanwhile, studies on the impact of equal rights to rent and purchase on the well-being of the renting and purchasing groups have mainly investigated whether owning housing property affects the quality of education for children [43]. The quality of education for children affects the well-being of residents [44]. Education rights have a positive effect on residents’ sense of happiness. Equal rights to rent and purchase can narrow the happiness gap between the renting and purchasing groups, and improve the level of well-being [26].

Thirdly, previous research has explored the internal logic and significance of implementing equal rights to rent and purchase at the level of compulsory education resources supply. They have explained the internal logic from two levels, namely, the logic of fairness and the logic of justice. On the one hand, the logic of fairness. Equal rights to rent and purchase is essentially a process of adjusting and distributing the public interest. The fair allocation of compulsory education resources is reflected in the fairness of education rights and access to education [45]. On the other hand, the logic of justice. Rawls regards justice as the primary value of social institutions [46]. The internal connection between housing and compulsory education is reflected in the absolute limit of the connection between the right to compulsory education and the household registration system [47]. Equal rights to rent and purchase reflects the justice value of the universality of compulsory education in the process of allocating compulsory education resources. In terms of theoretical value, the essence of equal rights to rent and purchase lies in delinking compulsory education resources from housing property rights [2]. Equal rights to rent and purchase is a beneficial and innovative breakthrough in alleviating the structural contradiction in the supply of compulsory education resources. In terms of practical significance, equal rights to rent and purchase alleviates the contradiction between the supply and diversified demand for compulsory education.

### 2.3 Factors and mechanisms influencing the supply of compulsory education resources in the context of equal rights to rent and purchase

To realize the balanced supply of compulsory education resources, the implementation of equal rights to rent and purchase has promoted the interactive symbiosis between educational space and social space to some extent. And this policy guidance is expected to become the driving force behind the effective interaction between education resources and social needs [48]. However, previous studies have mostly focused on the impact of equal rights to rent and purchase on the supply of compulsory education resources. They have not yet made a clear and in-depth explanation for the influencing factors and mechanisms of the supply of compulsory education resources in the context of equal rights to rent and purchase. This paper aims to fill such gap.

#### 2.3.1 Factors influencing the supply of compulsory education resources in the context of equal rights to rent and purchase

Equal rights to rent and purchase mainly affects the supply of compulsory education resources at two levels. Firstly, from the perspective of developing the housing rental market, equal rights to rent and purchase means that adjustments are made to the housing supply structure at the central level in China. It aims to guarantee the rights of tenants and promote equality between purchasers and tenants in their social rights and access to education resources [19]. Secondly, from the perspective of social well-being, the right to housing is the most basic requirement for people’s well-being and livelihood. The housing rental market carries the housing rights of many low and middle-income residents and floating population. Letting the housing rental market serve as a regulator for the allocation of compulsory education resources [49], and squeeze the right to survival with the right to development will undoubtedly amplify social inequality. In an ideal state, the right to housing and equal access to compulsory education resources cooperate should form a joint force. This will promote the development of the housing system and the balanced supply of compulsory education resources.

However, in practical situations, these two rights demonstrate the characteristics of separation, which are affected by many factors. The government is the main provider of compulsory education resources. The government bears the responsibility of reasonably decoupling the right to access compulsory education resources from the right to housing [50]. Firstly, the understanding of the supply of compulsory education resources is generally limited to technology embedding. The impact of technology on the supply of compulsory education resources has been a long-term concern. However, technology alone cannot improve the supply level of compulsory education resources. The efficiency of the supply of compulsory education resources is also affected by organizational innovation ability and social environment [51]. Secondly, some researchers suggest that the government has a flexible role in decoupling compulsory education resources from housing. In other words, to break the traditional linear supply model of compulsory education resources, the government does not have to be confined to its technological level [52]. Instead, the government should make full use of the advantages of information technology to form collaborative and sharing relationships amongst the main subjects [53]. Even when the technological level is limited, the collaborative supply platform built directly based on the demand for education can efficiently improve the quality and supply of compulsory education resources [54]. Finally, if equal rights to rent and purchase can work effectively and compulsory education resources can be shared through a collaborative supply platform, then synergistic effects may be realized. For example, if the government has a large amount of data for constructing a platform where two-way sharing of housing and compulsory education information can be realized, then they will generate the positive superposition effect of equal rights to rent and purchase and the supply of compulsory education resources [55]. However, due to the complexity of the social environment and policy implementation in China, equal rights to rent and purchase may have adverse effects, such as market structural contradictions and social welfare losses.

Existing research has pointed out that the balanced compulsory education resources supply in the context of equal rights to rent and purchase is mainly affected by the technological, organizational, and environmental dimensions. From the technological perspective, digital technology and platform construction are considered the core elements of the technological dimension [56]. It is represented by intelligent housing management platforms, digitization and informatization of education, and the construction of government cross-departmental information exchange platforms. From the organizational perspective, existing research suggests that the prerequisite for the effective implementation of equal rights to rent and purchase is as follows. It involves eliminating the institutional foundation of ‘emphasizing selling over renting’ [57], reconstructing the operating mechanism of equal rights to rent and purchase [58], Rather than the technological innovation alone [59, 60]. Apart from technological innovation, organizational innovation is also crucial to adapt to the new changes in the new era of the coordinated development of renting and purchasing, and to identify elements, such as government support, model innovation and human capital. Amongst these elements, government support includes financial resources, public policy and other factors. Positive government support can improve the efficiency of resource allocation and promote the innovation of the governance model. From the environmental perspective, the government can establish a governance system that focuses on the coordinated and balanced development of various public services through participatory governance. This governance system can also reform governance towards unifying the rights to housing and access to compulsory education resources [61]. The aim is to improve the governance environment [62]. Public policies with good value relevance and authority can accelerate the development of housing and compulsory education towards standardization, institutionalization and normalization [63].

#### 2.3.2 The role of the government and its influence mechanism on the supply of compulsory education resources in the context of equal rights to rent and purchase

The ‘National Medium and Long-term Education Reform and Development Plan (2010–2020)’ explicitly regards the promotion of fairness as a basic education policy direction. And it points out that the fundamental measure of promoting educational fairness is to allocate education resources reasonably and narrow the educational gap. The balanced compulsory education resources supply requires deepening the supply-side structural reform in compulsory education. The goal is to meet the public demand for quality education with the balanced allocation of compulsory education resources. As a result, the government faces urgent structural reform tasks, complex stakeholders and vague governance boundaries, which necessitate the innovation of governance paths. The concept of government governance in the context of equal rights to rent and purchase covers two aspects. They are the guarantee of the fundamental housing rights and the unification of guaranteeing the rights to housing and equal access to compulsory education resources. Under these two concepts, the governance role and its influence mechanism of the government in achieving the balanced compulsory education resources supply are heterogeneous.

Under the concept of ‘ensuing the right to housing’, the government mainly acts as a service provider. It aims to solve the unbalanced and inadequate development of the housing market and promote its optimization and upgrading by monitoring and tracking different housing demand groups [64]. Due to the understanding that the construction of housing digital platforms is confined to technological embedding, the role of the government as a service provider has been concerned for a long time. This type is referred to as government governance in the general sense, which relies on the promotion effect of the technology itself yet lacks systematic thinking at the level of government governance [65]. Nevertheless, local governments in China have promoted the construction of technological infrastructure (including data platforms) and strengthened the effectiveness of using technology in government governance. These measures have also contributed to the realization of the right to housing. This governance role mainly affects the supply of compulsory education resources through two sub-paths. One of these sub-paths directly improves the quality of compulsory education resources supply by driving public services through technology. In other words, the government comprehensively integrates and analyzes multiple heterogeneous data, such as housing, population and transportation. It aims to generate technological insights into the performance characteristics and matching efficiency between the supply and demand of housing and compulsory education. And on this basis, using these insights to achieve the precise supply of compulsory education resources [66]. Secondly, the government indirectly improves the quality of compulsory education resources supply by supervising and inspecting real estate developers, schools and other entities. In other words, the government uses information technology to efficiently manage real estate developers, schools and other participants. However, some studies have pointed out that technological governance may be restricted by certain factors, such as institutions, policies, talents and funds [67]. In particular, the blind use of technology may lead to inefficient factor allocation. It would deviate from its original intention of promoting the quality of compulsory education resource supply in the context of equal rights of renting and purchasing.

The concept of ‘ensuring equal access to compulsory education resources’ requires local governments to promote the reform of government governance concepts. The aim is to facilitate the utilization of technology in the supply of compulsory education resources, dealing with various complex problems effectively [68]. In recent years, the Chinese government has responded to the aforementioned problems by playing the role of a data platform builder. and related research has gradually received more attention. The platform acts as a government governance platform and a multi-subject collaborative supply platform that promotes information exchange, value co-creation and other model effects [69]. In other words, the goal of the platform is to facilitate the co-construction and sharing of compulsory education resources and improve the quality of compulsory education resource supply. This governance role mainly affects the supply of compulsory education resources through two sub-paths. Firstly, the government establishes a collaborative sharing platform that involves multiple entities, including itself, to integrate the elements of compulsory education resources [70]. For example, the government governance model shifts towards openness, democracy and information transparency. This shifting can break down those barriers that separate different levels and departments of the government, thus achieving the purpose of collecting, exchanging, integrating, processing, discovering, and utilizing information and resources. Secondly, faced with the fragmented compulsory education demands of the public, the government builds an interactive platform that connects its departments to the public. It aims to promote the formation of a differentiated compulsory education resources supply system with local characteristics [71]. In general, this data platform is an organizational structure formed by the government in response to the public demand to unify housing with compulsory education. This platform can significantly reduce the costs of cognition and communication amongst entities and achieve resource complementarity and coordinated development [72].

### 2.4 Review of the literature

Existing studies have mostly focused on theoretical analyses and policy commentaries. Existing studies have explored the development history and implementation status of equal rights to rent and purchase. These studies have also investigated the elements and mechanisms influencing the balanced compulsory education resources supply from the perspective of governmental governance in the context of equal rights to rent and purchase. It provided a solid foundation for this study and for future theoretical and empirical research. However, the construction of a theoretical framework for this topic faces several shortcomings. Firstly, rooted in Chinese practice, Firstly, the importance and priority of influencing factors have been disregarded in the literature. Thus it challenges the accurate identification of those key factors that influence the formation of differentiated driving models of the balanced compulsory education resources supply in China. Secondly, existing research has focused on the mechanism behind the influence of the government—as the main body of governance—on the supply of compulsory education resources. Ideally, all households should have equal access to compulsory education resources. However, in practice, the governance environment in China is highly complex, where many influencing factors drive the differentiated path of compulsory education resources supply through complex linkages. Existing studies have not systematically explored the complex interactive relationships amongst unique factors, such as technological levels, organizational capabilities and governance environments across different regions. It is still insufficient to provide adequate theoretical support for the diversified paths of compulsory education resource supply in the context of equal rights to rent and purchase. To fill these gaps, this paper will explore the key factors that influence the balanced compulsory education resources supply in the context of equal rights to rent and purchase and the differentiated driving paths of factor synergy.

## 3 Research framework

### 3.1 ‘Technology-organization-environment’ TOE framework

This study uses the TOE framework to identify those key factors affecting the balanced compulsory education resources supply in the context of equal rights to rent and purchase. The TOE framework is a comprehensive analysis framework for technological applications [73, 74]. This framework holds that the result of the technological application is influenced by three levels: technology, organization, and environment. The technological element focuses on the technology itself and its availability to organizations [75]. The organizational element emphasizes that technology cannot determine its own development process, cannot be utilized fully and rationally. The technology is subject to the intermediary influence of the organization [76]. For example, governments that pay more attention to technological innovation are more willing to construct service platforms, to fully release technological vitality. The environmental element refers to those variables to which technology may be exposed when operating externally [74], such as the institutional environment at the government level [75], the scale of public demand [77], and the level of economic development [78]. The TOE framework has been used to explore government governance [79]. The supply of compulsory education resources is a part of government governance. An effective synergy of the elements in the TOE framework promotes the formation of positive results [80]. While ineffective synergy of the elements of the TOE framework fails to produce positive results [81]. Therefore, this study adopts the TOE framework to investigate the complex interactions amongst various elements influencing the supply of compulsory education resources in the context of equal rights to rent and purchase.

### 3.2 Research method

The QCA method is a paradigm that analyzes the configuration effect of multiple elements from a holistic perspective. This method has been widely used in several management fields, such as public management [82], strategic management [83]. This study adopts the fsQCA method to explore how technological, organizational, and environmental variables affect the balanced compulsory education resources supply through linkage effects. Firstly, from the perspective of complex causality, the interdependent linkage of technological, organizational, and environmental elements affects the balanced compulsory education resources supply. However, based on reductionist assumptions, the traditional correlation theory models focus on simple symmetrical linear relationships between individual antecedents and outcomes. Instead of the complex causal relationships amongst multiple concurrent causes [84]. Due to the lack of correlation theory, and the mismatch between extant methods and complex phenomena. One of the most common mistakes in previous research is representing set relationships as correlation relationships and explaining the causal logic behind complex phenomena [85]. Based on the above issues, academics have gradually realized the necessity of breaking through the limitations of traditional correlation theories and methods. They have also developed the fsQCA method as a new research paradigm based on the configuration perspective. The fsQCA method views social phenomena holistically from the perspective of configuration. The fsQCA method studies the interdependence and interaction between conditions to collectively form multiple concurrent causes and equivalent paths leading to the occurrence of results [86]. It is suitable for studying the configuration paths of the linkage matching effects of technological, organizational, and environmental elements affecting the balanced compulsory education resources supply. It is in line with the research content of this study. Secondly, the application of the fsQCA method aims to identify the core influencing factors and configuration paths for achieving the balanced compulsory education resources supply in China as a whole and in eastern, central and western China. Multiple regression analysis method can only examine which factors influence the occurrence of the outcome variable. However, it cannot distinguish between core conditions and marginal conditions that influence the occurrence of the outcome variable. This study analyzes the core and marginal conditions that affect the balanced supply of compulsory education resources, and the configuration paths of antecedent conditions. The aim is to study the realization path of driving the balanced compulsory education resources supply. It is beneficial for revealing the configuration paths of the linkage matching effects of technological, organizational, and environmental conditions for achieving the balanced compulsory education resources supply. It is instructive in enriching the existing research.

The fsQCA method applies set theory and Boolean algebra to examine the relationship between conditions and outcomes from the perspective of sets. It uses Boolean algebra algorithms to formalize the logical process of analyzing the problem. Instead of emphasizing the ‘net effect’ of a single factor on the outcome, configuration theory and fsQCA method focus on complex phenomena in which multiple factors interact to generate the outcome. The fsQCA method focuses on the impact of the combination of multiple condition variables on the outcome variable. And it offers strong explanatory power for complex phenomena where multiple causes lead to a single outcome. Specifically, the fsQCA method can form a truth table using membership degree fuzzy sets. Then based on the truth table, it can be identified which combinations of causal characteristics generate the outcome characteristics. These combinations of causal characteristics are simplified through Boolean algebraic algorithms. This process primarily uses set relationships and the rules of logical operations between sets to explore the impact of multiple predetermined antecedent conditions on the outcome [87]. The fsQCA method is based on the clear-set qualitative comparative analysis method (csQCA). The fsQCA method converts the data into any value in the interval 0 to 1 to represent the data set, preventing data loss during the processing.

Based on the research question and specific research context, the fsQCA method is suitable for studying the impacting paths of the linkage effects of multiple factors on the balanced compulsory education resources supply. Neither technological, organizational, nor environmental factors can alone reveal the generation of the balanced compulsory education resources supply. While the fsQCA method is more suitable to study the multiple concurrent causalities of technological, organizational, and environmental factors affecting the balanced compulsory education resources supply. Therefore, the fsQCA method is aligned with the research question and research purpose.

In summary, this study is based on the element configuration generated by the fsQCA method. This study uses the fsQCA method to study two aspects. Firstly, previous studies have not clarified which technological, organizational, and environmental factors play a crucial role in the supply of compulsory education resources in the context of equal rights to rent and purchase. This paper clarifies the core elements by analyzing the existing research. Secondly, the ‘net effect’ analysis of a single factor cannot fully reveal which key factors affect the supply of compulsory education resources in the context of equal rights to rent and purchase [88]. By contrast, the configuration theory offers a holistic perspective. It conducts the comparative analysis at the case level, which is suitable for studying the impact of the combination of multiple factors on the outcome variable. Therefore, this paper selects 31 cities in China as case samples for the use of fuzzy-set qualitative comparative analysis. It aims to reveal differentiated supply models of compulsory education resources produced by the synergistic effects of technological, organizational, and environmental elements.

### 3.3 Case selection

The cases are selected in three steps. Firstly, urban administrative regions are taken as research objects. This is because cities are the most important housing market and areas with the most abundant education resources. The unique functions of resource circulation and factor agglomeration of a city make them important supports for housing and education development. Reforms in the housing and education sectors promoted by city governments are more common in education governance actions. Therefore, cities are the ideal objects of this study. Secondly, the study aims to explore the overall situation of equal rights to rent and purchase and the supply of compulsory education resources in China. Therefore, this study adopts the development examples nationwide. Due to the relatively low availability of data from Hong Kong, Macao, and Taiwan. Therefore, this study selects 31 provinces (autonomous regions and municipalities directly under the central government of China) in China, excluding Hong Kong, Macao, and Taiwan. And a typical city is chosen from each province as the research scope. The supply of compulsory education resources in the context of equal rights to rent and purchase requires a certain guarantee. And cities with more attention allocation and resource tilt from higher authorities are more likely to have the conditions to drive the balanced compulsory education supply. Therefore, the research focus is further narrowed to those cities where equal rights to rent and purchase has been more maturely implemented. Thirdly, the selection of cases mainly follows five principles. The first is the principle of homogeneity, which means all cases are comparable in technological, organizational, and environmental dimensions. The second is the principle of diversity, which means there is a certain degree of differentiation among the cases. The cases include both ‘effective’ and ‘ineffective’ results. The third is the principle of classicism, it involves selecting cases with a broader impact and are more typical of the situation. The fourth is the principle of the applicability of the selected antecedent conditions. The selected cases have distinguishable values in the antecedent conditions to ensure that these cases and the antecedent conditions are set to fit each other. The fifth is prioritizing these cases with more available research data to construct a case database. Combined with local practice, 31 analysis cases are ultimately selected.

In addition to field investigations, textual data in this study are collected from relevant policy documents and relevant literature that has been published or publicly available. As well as government work reports published on local government websites in China, relevant case news materials published by Chinese authoritative media (e.g. Xinhua News Agency and People’s Daily). For the same category of data in different literatures, the order of policy documents, official statistics, academic literature, and news material texts are used as the filter order, and the latest time is used as the basis for the secondary filter.

## 4 The influencing factors of the balanced compulsory education resources supply in the context of equal rights to rent and purchase

### 4.1 Condition analysis

According to Rihoux and Ragin, the number of configuration conditions in a medium-sized sample (10–40) should range from 4 to 7. This research identified six secondary elements, namely, data co-construction and sharing, technological infrastructure, attention allocation, government information disclosure, policy support for equal rights to rent and purchase and level of urban economic development.

#### 4.1.1 Technological conditions

Technological conditions include two secondary elements, namely, data co-construction and sharing and technological infrastructure. In the interaction between technology and organization, the characteristics of technology affect a series of behaviors such as the adoption and application of technology by organizations [82]. Based on transaction cost theory, data co-construction and sharing can effectively solve the problem of information asymmetry in government education governance. These two elements utilize the data platform to establish a solid government information disclosure mechanism, to regulate government behaviors and reduce information communication costs. The aim is to improve the efficiency of the supply of compulsory education resources [89, 90]. In actual government education governance, technological infrastructure is an essential foundation for driving the balanced compulsory education resources supply. The government strengthens the construction of technological infrastructure to facilitate information exchange, updates and optimizes the technological network, and builds an information resource network platform. Only in this way can the government ensures that technology plays an effective role in the supply of compulsory education resources. Thus, the supply of compulsory education resources can be sustainable.

#### 4.1.2 Organizational conditions

Organizational conditions include two secondary elements, namely, attention allocation and government information disclosure. Bounded rational decision theory holds that attention is a scarce resource. It considers that the behavioral choices of a decision maker depend on the issues and answers on which the decision maker is focused [91]. The policy agenda is the ‘gatekeeper’ of the government’s decision-making process. For example, why do some issues capture the attention of the government, while other issues lie off the government’s decision-making agenda? According to Simon’s bounded rational decision theory, attention driving decides government decision making. This paper focuses on the impact of organizational attention allocation on the supply of compulsory education resources. In the practice of e-government construction in China, a prominent problem is the relationship between e-government construction and government information disclosure [92]. The development of information technology provides a good platform and opportunity for government information disclosure in China. Information technology improves the sharing of government education information and gets rid of geographical restrictions. All citizens can use the education resource sharing platform to obtain government information. Information technology enhances the serviceability of education information disclosure, government credibility and improves the efficiency of compulsory education resources supply.

#### 4.1.3 Environmental conditions

Environmental conditions include two secondary elements, namely, policy support for equal rights of renting and purchasing and level of urban economic development. The environment is not directly involved in the process of education resources supply yet affects and dominates the interaction amongst the internal elements of the education system and the operation mode and the function of the whole system [93]. Resource dependence theory and innovation ecosystem theory discuss the synergistic relationship between the organization and the external environment from different perspectives. Such relationship is mainly reflected in environmental factors, such as the level of urban economic development and the intensity of policy support. The government supports education activities through policy support. The economic environment of a city can attract various resources, such as funds and teachers. Whether these resources are adequate or not greatly depends on the level of urban economic development, and such adequacy affects the implementation of education activities.

### 4.2 Model construction

**Fig 1:**
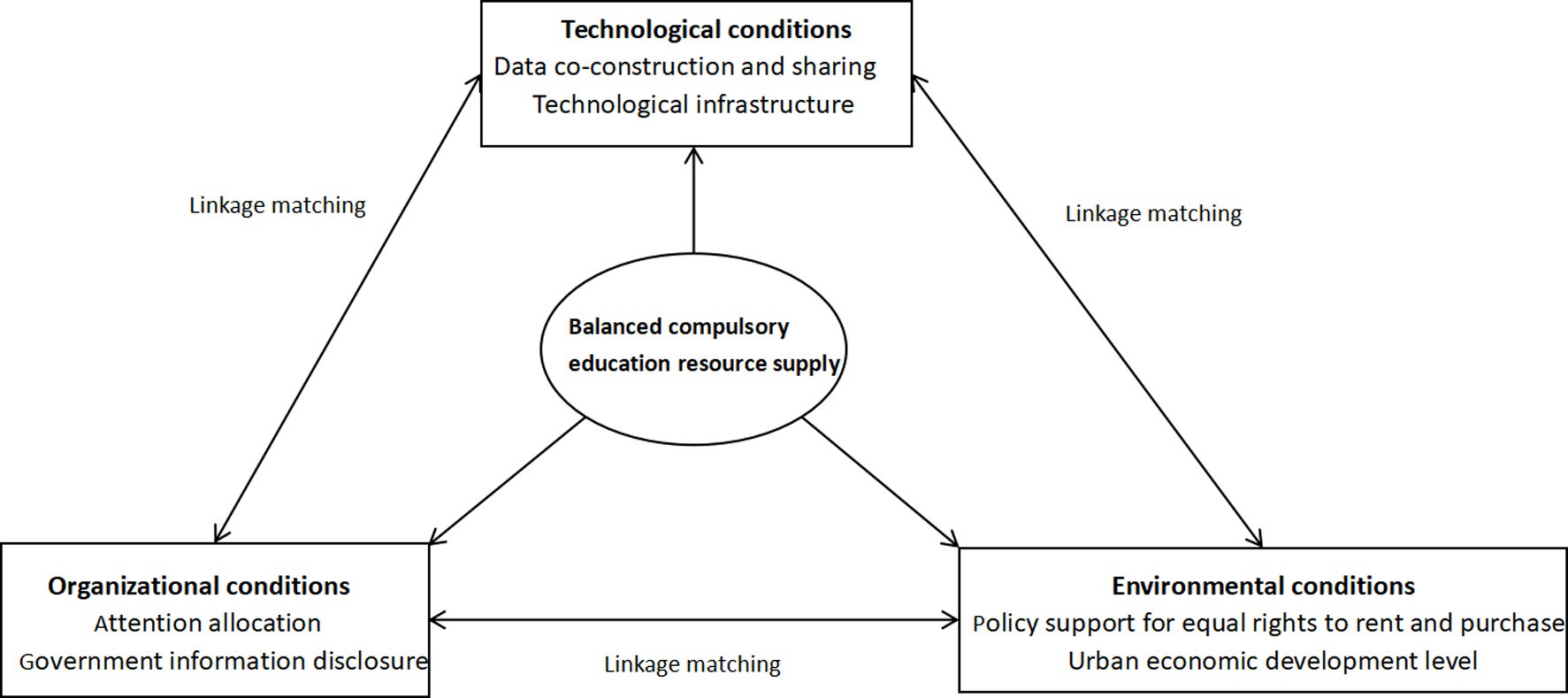
Balanced compulsory education resource supply model.

Given the above analysis, cities in China differ in terms of the performance of factors and factor coordination ways. The differentiated paths drive the balanced compulsory education resources supply. It can be seen that the perspective of the ‘net effect’ analysis of elements cannot sufficiently explain the complex causal mechanisms. However, the configuration perspective will help to solve this problem. This paper uses six secondary elements as the antecedent conditions for fsQCA analysis to construct a balanced compulsory education resource supply model (as shown in Fig 1).

## 5 The configuration of the balanced compulsory education resources supply in the context of equal rights to rent and purchase

### 5.1 Data construction

The fsQCA analysis uses the above samples and information. In addition, government documents (e.g. work reports and statistical yearbooks) and mainstream media reports (e.g. People’s Daily) are supplemented as needed. Triangular verification is formed by data from different ways to ensure data accuracy. The data construction of fsQCA includes two steps, namely, data measurement and data calibration. In the fsQCA method, each condition and the result is regarded as an independent set. Each case in these sets is assigned a membership score. The process of assigning a set membership score to a case is calibration [78]. Calibration is divided into direct calibration and indirect calibration [9]. Direct calibration relies on numerical settings associated with qualitative anchor points. Indirect calibration relies on researchers’ extensive grouping based on the membership degree of a case in the target set [94]. Moreover, the calibration should be guided by theory and the actual situation of a case, rather than mechanical calibration [89]. Based on the existing research, this paper uses the direct calibration method and the indirect calibration method. The purpose is calibrating the data according to the data type of the conditions and results based on the existing theoretical and empirical knowledge. For the specific variables determined by the real situation of the case, this paper adopts a case-oriented calibration method based on the actual situation of cases combined with qualitative data. The measurement and calibration results are shown in Table 1.

**Table 1:** Calibration of conditions and results.

Conditions and results	Measurement methods	Anchor points
Balanced compulsory education resources supply	Whether it is on the list of counties (cities, districts, and flags) that are first to be created for quality and balanced compulsory education nationwide in China	1, 0
Data co-construction and sharing	Whether interdepartmental data sharing is achieved	1, 0
Technological infrastructure	Number of internet broadband access users	8359250, 3484900, 815754.5
Attention allocation	Interval of issuance of local implementation documents by municipal governments in China	1.0, 0.8, 0.6, 0
Government information disclosure	Level of openness of government information	80.24, 66.83, 46.755
Policy support for equal rights to rent and purchase	Intensity of policy support for equal rights to rent and purchase	1, 0.67, 0.33, 0
Level of urban economic development	Urban economic development rating	1, 0.67, 0.33, 0

#### 5.1.1 Outcome variables

Balanced compulsory education resource supply. In order to realize the balanced development of compulsory education, ‘government fulfilling its duties’ plays a key role in realizing the basic balance and quality balance of compulsory education. All provinces in China have issued implementation measures and work plans for evaluating and identifying quality balanced development. And all provinces are formulating promotion plans considering their actual situation. Therefore, this study uses the degree of balanced development of compulsory education to measure the balanced compulsory education resources supply. In 2013, China officially launched the supervision, evaluation and certification of the basic balanced development of compulsory education and constructed an index system for evaluating compulsory education quality. Since then, compulsory education in China has entered the decisive stage. Compulsory education is moving from the decisive stage of achieving basic balance to a new stage of solidly promoting quality balanced development. By the end of December 2019, a total of 2767 counties nationwide in China had passed the basic balance identification of compulsory education, accounting for 95.32%. While 136 counties in 9 provinces in China had not passed the basic balance identification, including 39 counties in two provinces in central China and 97 counties in seven provinces in western China [95]. This result synthesizes those counties or cities that have passed the supervision, evaluation and recognition of the basic balanced development of compulsory education from 2013 to 2019. This result authoritatively measures the quality of compulsory education resources supply from the perspective of the Chinese government. Therefore, this paper uses the measurement method of whether counties or cities are on the list of counties (cities, districts, and flags) that are first to be created for quality and balanced compulsory education in China. This approach aims to measure whether these counties or cities have achieved the balanced compulsory education resources supply. All counties in a city that are on the list are assigned a value of 1, while those that are not listed partially or fully are assigned a value of 0.

#### 5.1.2 Condition variables

Technological conditions. This paper assigns a value to the condition of data co-construction and sharing based on whether the city has built an education data platform and realized data sharing through playing the role of the platform. If all these conditions hold true, then this city is assigned a value of 1; otherwise, this city is assigned a value of 0. However, unified data construction standards and norms are unavailable in various cities in China. Therefore, the research team uses in-depth interviews and qualitative evidence to determine whether a city has realized data co-construction and sharing. This study takes an important city located in Southwest China as an example. The head of the city’s education department stated, ‘We have established a data sharing mechanism with some public service departments, and we share some public data with other departments after desensitization.’ Such information can be used to determine whether the city has realized data co-construction and sharing or not. On the contrary, governments of some cities officially claimed that they have established a public service data center. However, after asking some probing questions, the interview results show that these data centers only present static data, which are not indicative of data co-construction and sharing. Technological infrastructure is measured following the criteria defined by Tan HB et al. Specifically, the number of internet broadband access users in each city in China in 2019 is used as an indicator to measure the level of technological infrastructure construction in a city. The data are collected from the China Statistical Yearbook (2020).

Organizational conditions. Attention allocation is measured following the method proposed by Tan HB et al. Local governments implement the policy guidelines set out by the central government of China by issuing local implementation opinions [91]. Therefore, taking the length of the time interval between the municipal governments issuing local documents on the implementation of equal rights to rent and purchase as the measurement method for attention allocation. This measurement method aims to indirectly measure the government’s degree of emphasizing the supply of compulsory education resources in the context of equal rights to rent and purchase. In this process, whether the policy has real enforcement power should be confirmed by combining interview data with second-hand information. Those policies that have yet to be implemented or have been abolished after short-term implementation should be excluded. According to the research of Chen X et al [94], the membership points are considered as follows. Firstly, the absence of policy documents can be considered as completely non-membership. Secondly, the issuance of one or more documents indicates that the government allocated some attention, it can be classified as partial membership. If the city had issued policy documents in each year from 2017, the year when the national proposal on equal rights to rent and purchase was first issued in China, to 2019, it can be classified as full membership. Rihoux and Ragin suggest that researchers can use different fuzzy sets based on actual knowledge and that equidistant intervals are not necessary across different levels [9]. Therefore, this study constructs a non-equidistant four-valued fuzzy set. The issuance of policy documents in all three years, issuance in two of the three years, issuance in one of the three years, and non-issuance of policy documents are calibrated as 1.0, 0.8, 0.6 and 0 respectively. No cases are considered as somewhat non-membership (0.4) and very non-membership (0.2). Government information disclosure is measured based on the China Government Transparency Index Report published by the Institute of Law at the Chinese Academy of Social Sciences in 2019. The average value of five indicators is used to determine the level of government information disclosure in each province. The five indicators and their respective proportions in the calculation are as follows: decision transparency (20%), management service transparency (25%), execution and results transparency (15%), critical area information disclosure (20%), and policy interpretation and response to concerns (20%).

Environmental conditions. According to Zhou ED et al, the strength and intensity of policy support for equal rights to rent and purchase are taken as the basis for measuring policy support for equal rights to rent and purchase [96]. Specifically, ‘the number of supporting policies in the city where the case is located’ is used to measure the strength and intensity of policy support for equal rights to rent and purchase. Taking into account the distribution characteristics of the selected case samples and referring to existing researches, this study adopts a four-value fuzzy set for anchor point division. Taking 10% percentile, 20% percentile, upper quartile as the first anchor point. Taking the average value, median, etc. as the second anchor point. And taking the 90% percentile, 80% percentile and lower quartile as the third anchor point [97]. The corresponding values of each case are converted to 0, 0.33, 0.67 and 1. Meanwhile, the grade of the city where the case is located is taken as the basis for measuring the level of urban economic development [98]. The fourth (fifth) tier, the third tier, the second tier, and the first tier are assigned to 0, 0.33, 0.67 and 1 respectively.

### 5.2 Analysis of necessary conditions

As the first step in fsQCA, necessary condition analysis mainly tests whether a certain condition is necessary for the occurrence of a result. This analysis provides the necessary support for subsequent sufficiency analysis. Consistency is an essential criterion for checking the necessary conditions. When the consistency is more than 0.9, the condition is considered necessary for the result. Table 2 shows the results of the necessary condition analyses for balanced and unbalanced supply of compulsory education resources as obtained from fsQCA software. It can be observed from Table 2 that the consistency levels of all conditions are less than 0.9. Therefore, there are no necessary conditions for producing a balanced or unbalanced supply of compulsory education resources. It is indispensable to further analyze the configuration of antecedent conditions.

**Table 2:** Analysis of necessary conditions.

	Balanced compulsory education resources supply		Unbalanced compulsory education resources supply	
**Condition variable**	**Consistency**	**Coverage**	**Consistency**	**Coverage**
High data co-construction and sharing	0.807692	0.840000	0.800000	0.160000
Non-high data co-construction and sharing	0.192308	0.833333	0.200000	0.166667
High technological infrastructure	0.522692	0.933379	0.194000	0.066621
Non-high technological infrastructure	0.477308	0.754866	0.806000	0.245134
High attention allocation	0.465385	0.801324	0.600000	0.198675
Non-high attention allocation	0.534615	0.874214	0.400000	0.125786
High government information disclosure	0.510385	0.930575	0.198000	0.069425
Non-high government information disclosure	0.489615	0.760454	0.802000	0.239546
High policy support for equal rights to rent and purchase	0.307308	0.774225	0.466000	0.225775
Non-high policy support for equal rights to rent and purchase	0.692692	0.870890	0.534000	0.129110
High level of urban economic development	0.668462	0.912815	0.332000	0.087185
Non-high level of urban economic development	0.331538	0.720736	0.668000	0.279264

### 5.3 Sufficiency analysis of conditional configurations

Configuration analysis aims to provide insights into the impact of different combinations of antecedent conditions on the results. Firstly, according to Schneider and Wagemann, the suitable threshold should be no less than 0.75 when assessing the consistency level of sufficiency [99]. Secondly, considering that the 31 cities selected in this paper are small- and medium-sized cities samples and that the researchers are familiar with all these cases, a low threshold of 1 is used [100]. Finally, PRI consistency is used to avoid the simultaneous existence of a subset relationship of configurations in both outcomes and non-outcomes. The value of PRI consistency should be close to the original consistency score [101]. Therefore, the PRI consistency threshold is set to 0.75. The results of the analysis are shown in Table 3.

**Table 3:** Configuration analysis of the balanced compulsory education resource supply in the context of equal rights to rent and purchase.

	‘Organization’	‘Technology–environment’	‘Technology–organization–environment’			‘Technology–organization’
Conditional configuration	Configuration 1	Configuration 2	Configuration 3	Configuration 4	Configuration 6	Configuration 5
Data co-construction and sharing		s			s	s
Technological infrastructure	x	S	s	S		x
Attention allocation	x	X	X	s	x	X
Government information disclosure	S		S	S	S	S
Policy support for equal rights to rent and purchase	X	x	x	s	x	X
Level of urban economic development	x	s	s	s	S	
Raw coverage	0.135000	0.228846	0.184231	0.191154	0.205769	0.112692
Unique coverage	0.032308	0.078846	0.012692	0.111154	0	0.008462
Consistency	0.980447	0.985099	0.985597	0.959459	0.987085	0.976667
Solution coverage	0.483846					
Solution consistency	0.982045					

Note: S and s indicate that the condition exists. X and x indicate that the condition does not exist. S indicates that the core condition exists. s indicates that the marginal condition exists. X indicates that the core condition is missing. x indicates that the marginal condition is missing. And blank indicates that the condition may or may not exist.

This study identifies six configurations of the balanced compulsory education resources supply. This study contributes to the configuration theory by interpreting these configurations through analyzing results and qualitative data and dialoguing with the theory. The consistency level of the six configurations in Table 3 for both individual and overall solutions is higher than the acceptable minimum standard of 0.75. The solution consistency is 0.982045 and the solution coverage is 0.483846. The six configurations in Table 3 are combinations of sufficient conditions of the balanced compulsory education resource supply.

Configuration 1 represents the ‘organization’ driving path. Configuration 1 shows the configuration path of promoting the balanced compulsory education resources supply when government information disclosure is the core condition. The consistency of this configuration is 0.980447. The unique coverage is 0.032308. The raw coverage is 0.135000. This configuration path can explain approximately 13.5% of all 31 cases. Additionally, this configuration path can only explain about 3.2% of all 31 cases. Configuration 1 is expressed as Balanced compulsory education resource supply = ~Technological infrastructure* ~Attention allocation* Government information disclosure* ~Policy support for equal rights to rent and purchase* ~Level of urban economic development* (* stands for the simultaneous existence of different conditions, and ~ stands for the absence of conditions). This formula indicates that when uncertainty is present in the condition of data co-construction and sharing, promoting government information disclosure can still generate the balanced compulsory education resource supply. The balanced supply can be achieved despite the absence of good technological infrastructure, high level of urban economic development, and effective government allocation attention and complete policy support for equal rights to rent and purchase. In other words, a high degree of government information disclosure can effectively break the constraints imposed by environmental, technological and other objective conditions on the ability of local governments to promote the balanced supply of compulsory education resources. Therefore, the condition of government information disclosure can alone constitute a sufficient condition for explaining the outcome. This configuration is named ‘organization’ driving path.

Configuration 2 represents the ‘technology–environment’ driving path. In Configuration 2, technological infrastructure is the core condition, data co-construction and sharing and level of urban economic development are the marginal conditions. The consistency of this configuration is 0.985099. The unique coverage is 0.078846. The raw coverage is 0.228846. This configuration path explains about 22.9% of all 31 cases. In addition, this configuration path can only explain about 7.9% of all 31 cases. Configuration 2 is expressed as Balanced compulsory education resource supply = Data co-construction and sharing* Technological infrastructure* ~Attention allocation* ~Policy support for equal rights to rent and purchase* Level of urban economic development*. This formula indicates that in addition to improving the level of urban economic development, promoting data co-construction and sharing and strengthening technological infrastructure also contribute to achieving the balanced compulsory education resources supply. The balanced supply can be achieved despite the uncertainty in government information disclosure. In this configuration, the existence of technological infrastructure condition plays a core role. Whilst data co-construction and sharing and level of urban economic development play complementary roles. Therefore, this configuration is named ‘technology-environment’ driving path.

Configurations 3, 4 and 6 represent the ‘technology–organization–environment’ driving paths. In Configuration 3, government information disclosure is the core condition, and technological infrastructure and level of urban economic development are the marginal conditions. The consistency of this configuration is 0.985597. The unique coverage is 0.012692, and the raw coverage is 0.184231. This configuration path explains about 18.4% of all 31 cases. In addition, this configuration path can explain about 1.3% of all 31 cases. Configuration 3 is expressed as Balanced compulsory education resource supply = Technological infrastructure* ~Attention allocation* Government information disclosure* ~Policy support for equal rights to rent and purchase* Level of urban economic development*. This formula indicates that with an orderly advancement of technological infrastructure construction and level of urban economic development, if the local government can pay more attention to and further strengthen the government information disclosure, then the local government can also promote the balanced supply of compulsory education resources. The balanced supply can be achieved despite there is uncertainty in the condition of data co-construction and sharing, and the lack of effective government attention allocation and complete policy support for equal rights to rent and purchase. In Configuration 4, technological infrastructure and government information disclosure are the core conditions. Attention allocation, policy support for equal rights to rent and purchase, and level of urban economic development are the marginal conditions. The consistency of this configuration is 0.959459. The unique coverage is 0.111154, and the raw coverage is 0.191154. This configuration path explains approximately 19.1% of all 31 cases. In addition, this configuration path can only explain about 11.1% of all 31 cases. Configuration 4 is expressed as Balanced compulsory education resource supply = Technological infrastructure* Attention allocation* Government information disclosure* Policy support for equal rights to rent and purchase* Level of urban economic development*. This formula indicates that good technological infrastructure, effective government attention allocation and government information disclosure, complete policy support for equal rights to rent and purchase and high level of urban economic development can also contribute to achieving the balanced compulsory education resource supply. The balanced supply can be achieved despite there is uncertainty in the condition of data co-construction and sharing. In other words, in addition to formulating and implementing more complete policies on equal rights to rent and purchase, the government should also strengthen the construction of technological infrastructure. The government also needs to improve its government information disclosure, its level of urban economic development and its attention allocation to balanced compulsory education resource supply. In Configuration 6, government information disclosure and level of urban economic development are the core conditions. Data co-construction and sharing is the marginal condition. The consistency of this configuration is 0.987085. The unique coverage is 0, and the raw coverage is 0.205769. This configuration path explains about 20.6% of all 31 cases. Configuration 6 is expressed as Balanced compulsory education resource supply = Data co-construction and sharing* ~Attention allocation* Government information disclosure* ~Policy support for equal rights to rent and purchase* Level of urban economic development*. This formula indicates that good data co-construction and sharing, better government information disclosure and high level of urban economic development contribute to achieving the balanced compulsory education resource supply. The balanced supply can be achieved despite the uncertainty in the technological infrastructure and the absence of effective government attention allocation and complete policy support for equal rights to rent and purchase. In other words, the government needs to improve the level of urban economic development and make continuous efforts in improving the transparency of government information and the level of data co-construction and sharing. In Configurations 3, 4 and 6, the government pays attention to the balanced compulsory education resource supply from technological, organizational, and environmental perspectives. Therefore, this type of configuration is called the ‘technology–organization–environment’ driving path.

Configuration 5 represents the ‘technology–organization’ driving path. Government information disclosure is the core condition. Data co-construction and sharing is the marginal condition. The consistency of this configuration is 0.976667. The unique coverage is 0.008462, and the raw coverage is 0.112692. This configuration path explains about 11.3% of all 31 cases. In addition, this configuration path can only explain about 0.8% of all 31 cases. Configuration 5 is expressed as Balanced compulsory education resource supply = Data co-construction and sharing* ~Technological infrastructure* ~Attention allocation* Government information disclosure* ~Policy support for equal rights to rent and purchase*. This formula indicates that better government information disclosure and data co-construction and sharing can facilitate the realization of the balanced compulsory education resource supply. The balanced supply can be achieved despite the lack of good technological infrastructure, effective government attention allocation and complete policy support for equal rights to rent and purchase. In other words, better government information disclosure and data co-construction and sharing conditions can break through the limitations of environmental conditions and drive the balanced compulsory education resource supply. As the driving path consists of the conditions of government information disclosure and data co-construction and sharing, it is named the ‘technology–organization’ driving path.

### 5.4 Analysis of the differentiated paths of compulsory education resources supply in eastern, central and western China

Influenced by economic development, geographical location, resource endowment and other factors, significant differences emerge in the supply of compulsory education resources across different regions of China. In addition, the differences in the institutional environment of different regions of China may also exert different impacts on the supply of compulsory education resources. As a result, these regions have differentiated paths for the supply of compulsory education resources in different regions of China. Therefore, based on the division criteria of eastern, central and western China according to the China Education Statistical Yearbook, this study further conducts a sub-regional configuration analysis.

Table 4 shows two configurations of compulsory education resources supply in the context of equal rights to rent and purchase in eastern China. Corresponding to Configuration 1 and Configuration 2, about 13.8% and 13.6% of all 31 cases can be explained respectively. Configuration 1 is expressed as Balanced compulsory education resource supply = ~Data sharing* ~Technological infrastructure* ~Attention allocation* ~Government information disclosure* ~Policy support for equal rights to rent and purchase*. This formula indicates that some cities in eastern China have the potential to achieve the balanced compulsory education resource supply. The balanced supply can be achieved despite the conditions of data co-construction and sharing, technological infrastructure, attention allocation, government information disclosure, and policy support for equal rights to rent and purchase are weak. Configuration 2 is expressed as Balanced compulsory education resource supply = Data co-construction and sharing* Technology infrastructure* ~Attention allocation* Government information disclosure* ~Policy support for equal rights to rent and purchase* Level of urban economic development*. This formula indicates that good technological infrastructure, government information disclosure and data co-construction and sharing will drive the balanced compulsory education resource supply. The balanced supply can be achieved despite the lack of complete policy support for equal rights to rent and purchase and insufficient government attention allocation. Eastern China is an economically developed region with good basic technological, organizational, and environmental conditions. These two configuration paths suggest that the combination of different conditions in eastern China can achieve the balanced compulsory education resources supply in a ‘different paths lead to the same destination’ manner.

**Table 4:** Configuration analysis of resource supply (balanced) for compulsory education in eastern, central and western China.

	Eastern China		Central China		Western China	
Conditional configuration	Configuration 1	Configuration 2	Configuration 3	Configuration 4	Configuration 5	Configuration 6
Data co-construction and sharing	x	s	s	s	S	S
Technological infrastructure	x	S	s	X		x
Attention allocation	x	X	X	s	x	x
Government information disclosure	x	s		x	x	
Policy support for equal rights to rent and purchase	X	x	X	s	x	x
Level of urban economic development		s	s	s	x	x
Raw coverage	0.138333	0.135833	0.210000	0.198571	0.262857	0.291429
Unique coverage	0.134167	0.095833	0.184286	0.182857	0.025714	0.085714
Consistency	1	1	0.967105	0.992857	0.948454	0.953271
Solution coverage	0.539167		0.67		0.63	
Solution consistency	1		0.989451		0.962882	

Note: S and s indicate that the condition exists. X and x indicate that the condition does not exist. S indicates that the core condition exists. s indicates that the marginal condition exists. X indicates that the core condition is missing. x indicates that the marginal condition is missing. And blank indicates that the condition may or may not exist.

There are two configurations of compulsory education resource supply in the context of equal rights to rent and purchase in central China. Corresponding to Configuration 3 and Configuration 4, about 21% and 19.9% of all 31 cases can be explained respectively. Configuration 3 is expressed as Balanced compulsory education resource supply = Data co-construction and sharing* Technological infrastructure* ~Attention allocation* ~Policy support for equal rights to rent and purchase* Level of urban economic development*. This formula indicates that some cities in central China are able to drive the balanced compulsory education resource supply by virtue of good data co-construction and sharing, improved technological infrastructures and a high level of urban economic development. The balanced supply can be achieved despite the uncertainty in government information disclosure and lack of good governmental attention allocation and complete policy support for equal rights to rent and purchase. Configuration 4 is expressed as Balanced compulsory education resource supply = Data co-construction and sharing* ~Technological infrastructure* Attention allocation* ~Government information disclosure* Policy support for equal rights to rent and purchase* Level of urban economic development*. This formula shows that improving the level of urban economic development, data co-construction and sharing and government attention allocation and establishing a complete policy support system for equal rights to rent and purchase can also drive the balanced supply. The balanced supply can be achieved despite the absence of good technological infrastructure and government information disclosure. It can be further analyzed through the analysis of the configuration in depth. Central China is located close to some developed provinces in eastern China. Central China has many geographic advantages. Compared with western China, central China is more likely to be influenced and radiated by the advanced technological conditions, organizational strategies and favorable political and economic environment of eastern China. Thus central China is located in a more advantageous position than western China. These five factors mentioned above are the core factors for the balanced compulsory education resources supply in central China. The five factors are data co-construction and sharing, technological infrastructure, attention allocation, policy support for equal rights to rent and purchase and the level of urban economic development. The different combinations of core conditions and marginal conditions can drive the balanced compulsory education resources supply in a ‘different paths lead to the same destination’ manner in central China.

There are two configurations of compulsory education resource supply in the context of equal rights to rent and purchase in western China. Corresponding to Configuration 5 and Configuration 6, about 26.3% and 29.1% of all 31cases can be explained respectively. Configuration 5 is expressed as Balanced compulsory education resource supply = Data co-construction and sharing* ~Attention allocation* ~Government information disclosure* ~Policy support for equal rights to rent and purchase* ~Level of urban economic development*. Configuration 6 is expressed as Balanced compulsory education resource supply = Data co-construction and sharing* ~Technological infrastructure* ~Attention allocation* ~Government information disclosure* ~Policy support for equal rights to rent and purchase* ~Level of urban economic development*. It can be seen that data co-construction and sharing is the core condition in both configurations. Compared with eastern and central China, western China does not have advantages at the technological, organizational and environmental aspects. In terms of driving the balanced compulsory education resources supply in western China, improving the level of urban economic development is the foundation. On this basis, the balanced supply can be achieved through a way of strengthening the technological infrastructure, raising the government attention allocation and enhancing the policy support efforts. Another way of achieving the balanced supply of compulsory education resources is raising the government attention allocation and strengthening the transparency of government information.

In summary, eastern, central and western China are affected by human capital, capital stock and degree of implementation of equal rights to rent and purchase. As a result, eastern, central and western China have differentiated driving paths for the balanced supply of compulsory education resources in the context of equal rights to rent and purchase.**5.5 Robustness test**

A robustness test is then performed on the above configuration paths. The QCA method is a set-theoretic approach. When slight changes are made to the operation, the newly generated configuration results show a clear subset relationship with the original configuration results but do not change the substantive interpretation of the research results [102]. In this case, these results are deemed robust. Based on this idea, this study uses the method of adjusting the consistency threshold of sufficiency and adjusting the consistency threshold of PRI for the robustness test. Firstly, the consistency threshold of sufficiency was increased from 0.8 to 0.85 [99], which produced new configurations that are consistent with the original results. Secondly, by reducing the PRI consistency threshold by 0.05 [103], that is, from 0.75 to 0.7. It was found that there was a subset relationship between the configurations under the two PRI consistencies. The new configuration is consistent with the original configuration results, and there was no significant change in the overall consistency and coverage. Therefore, the findings of this study are robust.

## 6 Conclusions and policies

### 6.1 Research conclusions

This paper used the fsQCA method for the first time to conduct a configuration analysis on samples of compulsory education resource supply cases in 31 cities in China in the context of equal rights to rent and purchase. This study specifically explores the linkage effects and driving paths of the impact of technological, organizational, and environmental factors on the supply of compulsory education resources. This study also elucidates the core conditions and their complex interactions that influence compulsory education resources supply in the context of equal rights to rent and purchase. The conclusions are summarized as follows.

Firstly, in general, technological, organizational, and environmental factors cannot individually serve as necessary conditions for the balanced compulsory education resource supply. It indicates that individual elements do not constitute the necessary antecedents for the balanced compulsory education resources supply. Six configuration paths are identified in this study, which correspond to four driving models for the balanced compulsory education resources supply. Specifically, it can be categorized as ‘organization’, ‘technology–environment’, ‘technology–organization–environment’ and ‘technology–organization’ driving models. The ‘organization’ driving model requires the government to constantly improve the transparency of its information and enhance its credibility to drive the balanced supply of compulsory education resources. The ‘technology–environment’ driving model requires the government to constantly improve the level of governance in terms of technological infrastructure, data co-construction and sharing, and other technological aspects. The ‘technology–organization–environment’ driving model requires the government to pay attention to the balanced compulsory education resources supply at the technological, organizational, and environmental levels. This driving model also requires the government to make sustained efforts in policy support, attention allocation, government information disclosure and other aspects. The ‘technology–organization’ driving model requires the government to continuously improve the transparency of government information and the technological level of data co-construction and sharing.

Secondly, due to the affection of economic development, resource endowment and other factors, there are differentiated paths of compulsory education resource supply in eastern, central and western China. Firstly, compared with central and western China, eastern China is an economically developed region with better technological, organizational, and environmental conditions. The two configuration paths in eastern China suggest that the combination of different conditions can achieve the balanced supply of compulsory education resources in a ‘different paths lead to the same destination’ manner. Therefore, eastern China should consistently build on its strengths on the basis of its good basic conditions. Eastern China also should maintain sustainable in promoting the balanced supply of compulsory education resources. Secondly, the two configuration paths in central China show that different combinations of core and marginal conditions can drive the balanced supply in a ‘different paths lead to the same destination’ manner. Local governments in central China should constantly improve the level and quality of governance at the technological, organizational and environmental levels and learn from the advanced experiences of some cities in eastern China. Thirdly, the two configuration paths in western China indicate that data co-construction and sharing is the core condition. Therefore, local governments in western China should improve the level of urban economic development. On this basis, the balanced supply can be achieved through a way of strengthening the technological infrastructure, raising the government attention allocation and enhancing the policy support efforts. Another way of achieving the balanced supply of compulsory education resources is by raising the government attention allocation and strengthening the transparency of government information.

### 6.2 Theoretical contributions

This paper makes the following theoretical contributions. Firstly, existing studies have explored the supply of compulsory education resources in the context of equal rights to rent and purchase. However, most of them have focused on theoretical analyses and policy commentaries [104]. Those key factors that influence compulsory education resources supply in the context of equal rights to rent and purchase have received scant attention. This paper fills such research gap to some extent. This paper is based on 31 cities in 31 provinces (autonomous regions and municipalities directly under the central government of China) where equal rights to rent and purchase have a mature implementation. From the perspective of ‘technology–organization–environment’, this paper identifies six key factors that affect the balanced compulsory education supply in the context of equal rights to rent and purchase. After the analysis of 31 cases, concrete influencing factors were obtained. Firstly, none of the technological, organizational and environmental factors are necessary conditions for achieving the balanced compulsory education resources supply. Instead, these factors should cooperate effectively to exert a significant effect. This finding lays the foundation for constructing the research model. Secondly, attention allocation and government information disclosure are the key organizational factors. The former determines the development direction of governance and strength of governance. While the latter is directly related to the credibility of governance. Existing research has mainly focused on static organizational capabilities, with an emphasis on the formulation and implementation of policies by organizations and the impact of these policies on present phenomena [105]. This study found that a breakthrough in organizational capability has been achieved in the practice of compulsory education resources supply in the context of equal rights to rent and purchase. Organizations can realize information interoperability and connectivity through their use of technology. These organizations can also enhance their governance capabilities and make better governance decisions by using dynamic information. Based on this, organizations have achieved breakthroughs in terms of their organizational capabilities. Given that existing research has not yet explored the use of technology by organizations characterized by organizational capability breakthroughs and the integration of technology and organization, the above finding represents a key innovation of this paper. Furthermore, the balanced compulsory education resources supply is also affected by environmental factors such as the policy support for equal rights to rent and purchase and the level of urban economic development. However, most of the existing studies remain at the level of the simple description, and have not yet conducted in-depth empirical research and discussion. In this study, it is found that technological, organizational and environmental factors have varying degrees of impact on compulsory education resources supply. The different configurations formed by the collaboration of these factors drive the balanced supply in a ‘different paths lead to the same destination’ manner. This finding also proves the reliability of the research model constructed in this study. To a certain extent, it provides a reference for future research on compulsory education resources supply in the context of equal rights to rent and purchase.

Secondly, compulsory education resources supply in the context of equal rights to rent and purchase is a complex system formed by many non-linear couplings. The supply of compulsory education resources is synergistically affected by differentiated factors, which in turn affects the government’s choice of governance model. The existing literature has not yet answered the question of which differentiated paths drive the balanced compulsory education resource supply by the Chinese government. This paper fills this research gap to some extent. This paper is based on 31 cities in China in which equal rights to rent and purchase have been carried out more maturely. Taking advantage of the straightforward case studies of the configuration approach, this paper identifies four driving models that generate the balanced compulsory education resources supply. Specifically: (1) It has been pointed out that the government, as a policy maker and promoter, influences the balanced compulsory education resources supply. This study further develops two findings. Firstly, when all factors in the technological, organizational and environmental levels perform well, a virtuous circle of ‘good performance of factors–driving the balanced supply of compulsory education resources’ is formed. It will provide conditions for the sustainable enhancement of government governance capabilities. Secondly, when one of the technological, organizational and environmental levels works alone or two levels work jointly, the government will strive to drive the balanced supply as much as possible by leveraging existing advantages. These findings add potential benefits for future research. (2) Government governance capabilities, policy support, and other governance conditions are different in eastern, central and western China. These conditions forms different governance structures. However, existing studies have not yet explained this heterogeneous phenomenon. This study provides an explanation for this heterogeneous phenomenon. This paper finds that influenced by regional differences in technological, organizational and environmental conditions, eastern China has better basic conditions at the technological, organizational and environmental levels. The combination of different conditions in eastern China can drive the balanced supply of compulsory education resources in a ‘different paths lead to the same destination’ manner. The development of central China is limited to some extent. It is still able to drive the balanced supply with different combinations of core and marginal conditions, due to the radiation and driving effect of eastern China. Western China lacks advantages at the technological, organizational and environmental levels compared to central and eastern China. And when organizational and environmental conditions are limited, the government will develop technological conditions to drive the balanced supply of compulsory education resources.

The above findings have expanded the insight and understanding of the core influencing factors and their complex interactions as well as configuration paths of the balanced compulsory education resources supply in the context of equal rights to rent and purchase. From multiple aspects of technology, organization, and environment, multiple driving models such as ‘organization’, ‘technology–environment’, ‘technology–organization’, ‘technology–organization’, ‘technology–organization–environment’ driving models, as well as multiple regions of eastern, central and western China.

### 6.3 Practical implication

This paper is based on the key influencing factors and the analysis of configuration model of compulsory education resource supply in the context of equal rights to rent and purchase in 31 cities nationwide in China. It attempts to provide governance path references for Chinese local governments. Chinese local governments can refer to development models with similar configurations, choose appropriate development directions, and cultivate development models that adapt to local characteristics. Based on the conclusions of this study, two policy recommendations are proposed.

Firstly, Chinese local governments should strengthen the synergistic integration of technological, organizational, and environmental factors. According to local characteristics, Chinese local governments should holistically focus on the linkage matching between technology, organization, and environmental conditions. Local governments should formulate policies to improve the supply level of compulsory education resources. Secondly, local governments should resolve the contradiction between the right to housing and equal access to compulsory education resources in the context of equal rights to rent and purchase. The highlight of equal rights to rent and purchase lies in the "equal rights" thinking. Before this, the absence of "equal rights" to rent and purchase stems from the scarcity of educational resources in China, making it difficult to meet every family’s demand for quality education resources. Another reason is the unreasonable thinking of government governance which is accustomed to treating people differently according to their types. Due to these issues, equal rights to rent and purchase is an inevitable requirement in modern public governance. However, in practice, to fundamentally achieve equal rights to rent and purchase, the government should increase the quantity and improve the quality of education resources supply further. For example, in the short term, the system can be designed to compress the space for speculation in the housing rental market. It is necessary to make corresponding restrictions on the right to rent out a house. For example, for the school district housing, it can be limited to those who do not own other houses in the city, including self-owned and rental housing. It aims to allow them to enrollment through rental housing in the district. And no vacancy or sublease is allowed during the rental period, and the minimum rental period for school district housing should be set. Through reasonable restrictions on rental conditions and behaviors, avoiding conflicts between the right to housing and equal access to educational resources, and the phenomenon of purely renting for "rights". In the medium and long term, the government in China should increase the investment in the supply of compulsory education resources. Especially, the Chinese government should provide more financial support for compulsory education in cities with net population inflows. Furthermore, the reform of equalizing basic public services should continue to be pushed forward. In particular, it is necessary to explore paths for realizing the balanced compulsory education resources supply. The Chinese government should also explore the reform of the household registration system, and coordinate the reform of the housing system with the reform of the supporting system simultaneously.
